# Over 10% of Surgically Treated High-Energy Pelvic Fractures Are Associated with Undiagnosed Ligamentous Knee Injuries: An Epidemiologic Study in Italy’s Largest Trauma Center

**DOI:** 10.3390/medsci13030124

**Published:** 2025-08-12

**Authors:** Simone Giusti, Vittorio Alfonsi, Edoardo De Fenu, Claudia Franco, Stefano Cacciatore, Francesco Liuzza, Ezio Adriani

**Affiliations:** 1 Complex Operational Unit of Sports Medicine and Joint Reconstruction, Fondazione Policlinico Universitario Agostino Gemelli IRCCS, 00136 Rome, Italy; 2 Department of Geriatrics, Orthopaedics, Rheumatology, Università Cattolica del Sacro Cuore, 20123 Milano, Italy; 3 Unit of Orthopaedic and Trauma Surgery, Policlinico Tor Vergata, 00133 Rome, Italy

**Keywords:** pelvic fractures, multi-ligament injury, ACL, PCL, undiagnosed

## Abstract

Purpose: To evaluate the prevalence of undiagnosed ligamentous knee injuries in patients surgically treated for high-energy pelvic ring or acetabular fractures and propose a mechanism to diagnose these briefly post-hospital discharge. Methods: A retrospective case series (level of evidence IV) was conducted at Italy’s largest trauma center. Medical records from 2018 to 2023 were reviewed to identify patients who underwent surgical treatment for pelvic or acetabular fractures. Eligible patients were contacted for a structured telephone interview, which included a questionnaire on knee symptoms and the International Knee Documentation Committee (IKDC) score. Associations between demographic factors, trauma mechanism, and knee outcomes were statistically analyzed. Results: Fifty-nine patients (mean age 55 years, 72.9% male) were enrolled. Undiagnosed knee ligament injuries were present in 11.9%, with an additional 8.5% reporting persistent knee symptoms. The average time to diagnosis was 6.4 months post-discharge. Patients involved in road traffic accidents showed a significantly higher incidence of knee injuries (34.8%) compared to those who fell from a height (3.9%) (*p* = 0.049). Patients who had undergone ligament reconstruction had significantly lower IKDC scores (62.0 ± 8.2) than non-surgical cases (82.4 ± 12.1, *p* = 0.0002). No association was found with age or sex. Conclusions: Ligamentous knee injuries are frequently overlooked in the acute management of high-energy pelvic fractures, particularly in road traffic accidents. A systematic knee assessment before discharge or early outpatient imaging should be considered to improve detection and outcomes.

## 1. Introduction

High-energy trauma, including motor vehicle accidents and falls from height, are amongst the primary causes of multi-organ injury [1,2]. Pelvic fractures (PT) commonly arise as a consequence of these high-impact accidents and are frequently associated with isolated or multi-ligamentous injuries around the knee joint [3,4].

The incidence of PT accounts for 5% of all skeletal injuries, with a significant prevalence among young adults presenting high Injury Severity Scores (ISSs) [5]. The acetabulum is the most commonly affected anatomical structure, with a frequency of 3 cases per 100,000 individuals, predominantly young adults aged 20 to 50 years [6]. The mortality rate for these injuries is significantly elevated (8–15%) [7], particularly in hemodynamically unstable patients, due to rapid blood loss and challenges in achieving hemostasis [7,8].

Given the high number of significant comorbidities, initial management typically prioritizes hemodynamic stabilization, which may occasionally lead to the neglect of secondary injuries, particularly capsuloligamentous injuries around the knee. These injuries can present with subtle clinical signs, especially when masked by altered consciousness, distracting injuries, or immobilization protocols. Therefore, a high index of suspicion is necessary when evaluating trauma patients with pelvic fractures, particularly those who are unable to provide reliable clinical feedback.

The anterior cruciate ligament (ACL) and posterior cruciate ligament (PCL) are the most frequently compromised structures [9,10,11]. As evidenced in the literature, early diagnosis and thorough management of knee ligamentous injuries is crucial to achieve high functional outcomes in the medium-to-long term and preventing the development of post-traumatic osteoarthritis and instability. To avoid these complications, it is essential to implement procedures that facilitate prompt diagnosis and timely management [12,13,14]. Identifying risk factors based on injury models or mechanisms could be pivotal for early diagnosis and treatment, ultimately aiding in the prevention of long-term complications [15].

The association between pelvic fractures and knee ligament injuries is likely influenced by the biomechanical forces transmitted through the lower limb during high-energy trauma. For instance, road traffic accidents often involve complex force vectors, including axial loading and rotational stresses, which can simultaneously affect the pelvis and knee. In contrast, falls from height may produce more localized axial compression, potentially sparing the knee joint. Understanding these biomechanical patterns is critical for developing targeted diagnostic protocols. Furthermore, the systemic inflammatory response triggered by polytrauma may mask early symptoms of knee injuries, as pain and swelling may be attributed to the broader traumatic insult rather than a specific ligamentous pathology. This underscores the need for a high index of suspicion and standardized evaluation protocols to ensure that knee injuries are not overlooked in the acute trauma setting.

This study aims to identify the percentage of undiagnosed knee ligament injuries in patients admitted with pelvic ring or acetabular fractures managed surgically in Italy’s largest trauma center, in order to provide a measure of how significant the issue is and what procedures could be put into place to help reduce the chance of these injuries being missed in the acute setting. By quantifying the prevalence of these injuries and exploring their association with trauma mechanisms, this study seeks to inform clinical practice and highlight the importance of systematic lower limb assessments in polytrauma patients. The findings may guide the development of diagnostic algorithms that prioritize early detection, ultimately improving functional outcomes and reducing the long-term burden of undiagnosed injuries.

## 2. Materials and Methods

Experimental protocol ethical approval was obtained by our institution’s ethics committee. The study was conducted according to the Helsinki Declaration and in adherence with human rights laws. We conducted a retrospective case series (level of evidence IV) at Italy’s largest trauma center. A retrospective medical record review was performed to identify all patients admitted with a pelvic ring or acetabular fracture treated surgically from 1 January 2018, to 31 December 2023, to allow for at least 12 months’ follow-up. The records were consulted by 3 different members of staff. To ensure accuracy and consistency, each reviewer independently screened the records using predefined inclusion criteria, which included a confirmed diagnosis of pelvic ring or acetabular fracture and surgical intervention. Discrepancies between reviewers were resolved through consensus discussions to minimize selection bias. Patients with incomplete medical records, those under 16 years of age, or those with pre-existing knee conditions unrelated to the trauma were excluded to focus specifically on trauma-related ligamentous injuries. This rigorous selection process aimed to enhance the reliability of the study cohort. An attempt was made to contact all of these patients via telephone call. Informed consent was obtained both verbally and in written form from all the participants who we were able to enroll. A verbal questionnaire was administered to all of the patients in order to identify if they had developed any knee pathology during or after their hospital stay (Table 1).

The questionnaire was designed to capture both acute and delayed-onset knee symptoms, focusing on indicators such as pain, instability, and reduced range of motion, which are suggestive of ligamentous injury. To standardize responses and reduce recall bias, the questionnaire was structured with closed-ended questions, supplemented by open-ended prompts to allow patients to elaborate on their symptoms. The telephone interviews were conducted by trained orthopedic residents to ensure consistency in data collection. Each interview lasted approximately 15–20 min, allowing sufficient time to gather detailed information while minimizing patient fatigue. For patients who reported knee symptoms, additional questions explored the timing of symptom onset, diagnostic evaluations performed post-discharge, and any subsequent treatments received. This approach enabled a comprehensive assessment of the natural history of knee injuries in this population.

**Figure 1:**
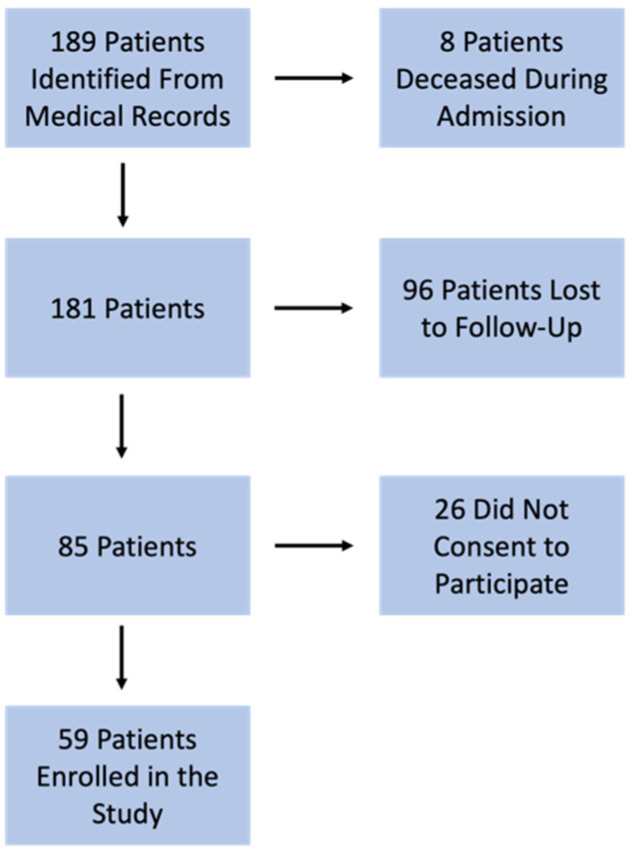
Participant selection flowchart.

All responding patients were also asked to complete an IKDC questionnaire, which was also filed via telephone consultation. The IKDC questionnaire was selected for its validated ability to assess knee function and symptoms in both surgical and non-surgical populations. A selection flowchart is provided in Figure 1.

## 3. Study Outcomes

**Figure 2:**
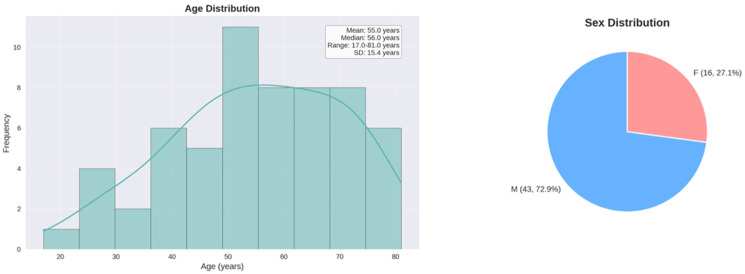
Age and sex distribution of the study population.

Demographic patient data, such as age at time of injury, sex, body mass index (BMI) and any relevant co-morbidities, were extrapolated from the medical charts. Patients who had received prior diagnosis or treatment for a ligamentous knee injury were identified from the records. Patient demographics are summarized in Table 2 and Figure 2.

## 4. Statistical Analysis

Statistical analyses were performed using IBM SPSS Statistics for Windows, Version 28.0 (IBM Corp., Armonk, NY, USA). Data of continuous variables were presented as mean values ± standard deviation (SD). Differences were considered significant at the *p* < 0.05 level. To evaluate associations between variables, we employed chi-square tests for categorical data (e.g., trauma mechanism and presence of knee injury) and independent *t*-tests for continuous data (e.g., IKDC scores between surgical and non-surgical groups). Logistic regression analysis was used to assess the influence of demographic factors (age, sex, BMI) and trauma mechanism on the likelihood of knee ligament injury. Normality of data distribution was confirmed using the Shapiro–Wilk test, and non-parametric tests were applied where appropriate. A power analysis was conducted to ensure the sample size was sufficient to detect clinically meaningful differences in knee injury prevalence, with an alpha of 0.05 and a power of 80%.

## 5. Results

The datasets used and/or analyzed during the current study are available from the corresponding author on reasonable request. The study population age ranged from 17 to 81 years, with a mean age of 55 years, a median of 56 years, and a standard deviation of 15.36. Out of 59 patients, the majority, 43 patients (72.88%), were male, and 16 (27.12%) were female. Among 59 patients, 20.34% currently have knee pathology and 18.64% experienced knee issues in the first 12 months post-discharge. The most commonly reported knee symptoms were joint instability, arthrofibrosis, and pain. Seven patients (11.86%) had undergone ACL or PCL reconstruction for a ligamentous injury sustained during the original pelvic trauma at the time of the telephone consultation. The average time from hospital discharge to diagnosis of knee ligamentous injury was 6.38 months from the traumatic event. We found no statistically significant associations between sex (*p* = 0.202) and age (*p* = 0.549) and likelihood of sustaining a ligamentous knee injury. However, a statistically significant association was identified between trauma mechanism and development of knee pathology (*p* = 0.049), with road accident-related trauma showing higher rates of knee injuries (34.78%) compared to falls from heights (3.85%). This strongly suggests that trauma mechanism may be an important predictor of long-term knee complications in patients admitted with pelvic ring or acetabular fracture.

The mean IKDC score amongst the 59 patients we interviewed was 79.93 (44–93 years, ±13.43). Patients who had received either ACL or PCL reconstruction had significantly lower IKDS scores compared to those who did not. In our analysis, these patients (7) showed a mean IKDC score of 62.00 (±8.23) compared to a mean score of 82.35 (±12.13) in the non-reconstructed group (52), *p* = 0.0002. There was no statistically significant correlation between IKDC score and trauma mechanism of the original pelvic-ring or acetabular fracture.

## 6. Discussion

In our study we aimed to investigate the prevalence of undiagnosed knee ligament injuries in patients undergoing surgical treatment for high-energy pelvic ring and acetabular fractures. The coexistence of major skeletal injuries, such as pelvic and acetabular fractures resulting from high-energy trauma (e.g., motor vehicle accidents or falls from significant heights), is often associated with other significant injuries [16,17]. It has been reported that up to 90% of patients with pelvic and acetabular fractures present with associated injuries such as thoracic injuries (10–21%), abdominal injuries (8–28%), urogenital injuries (3–22%), and lower limb injuries (35–41%) [16,18]. Our study adds another dimension to this complexity, suggesting that knee ligament injuries could be an underappreciated associated injury in this patient population [19,20].

The complexity of these clinical scenarios is dictated by the traumatic mechanism, which influences the type of fracture and the associated soft tissue damage. Mixed trauma, both contusive and distortive, involves the anterior cruciate ligament (ACL), posterior cruciate ligament (PCL), collateral ligaments (MCL and LCL), and often, the articular capsule itself. These factors, along with the potential presence of meniscal injuries and bone quality related to age, significantly affects clinical and radiographic outcomes, favoring the development of post-traumatic osteoarthritis.

The biomechanical forces involved in high-energy trauma provide further insight into why knee ligament injuries may occur concomitantly with pelvic fractures. Road traffic accidents, which were associated with a higher incidence of knee injuries in our study (34.78%), often involve complex multiplanar forces, including axial loading, shear, and rotational stresses. These forces can propagate through the lower limb, affecting the knee joint in addition to the pelvis. For example, a dashboard injury in a motor vehicle accident may cause posterior translation of the tibia, potentially injuring the PCL, while simultaneous rotational forces may compromise the ACL or collateral ligaments. In contrast, falls from height typically result in vertical compression forces, which may spare the knee joint unless significant angulation or rotation occurs upon impact. These differences in injury mechanics highlight the importance of tailoring diagnostic approaches to the trauma mechanism.

In addition, knee ligamentous injuries may not only be overlooked in the acute trauma setting but may also be misattributed as transient soft tissue injury. This diagnostic ambiguity may delay appropriate referrals or follow-up investigations. In our cohort, several patients only received a definitive diagnosis following the emergence of persistent functional complaints during rehabilitation. Another contributing factor to missed diagnoses may include resource limitations or prioritization of imaging for life-threatening injuries. Consequently, reliance on clinical signs alone, which may be obscured by pain or reduced consciousness, may result in underdiagnosis.

Among the 59 patients examined, 20.34% are currently affected by knee pathology, and 18.64% have experienced knee-related issues after discharge. The most common issues reported were joint instability (3 patients), joint rigidity (2 patients), and pain (2 patients).

Seven patients (11.86%) underwent anterior or posterior cruciate ligament reconstruction. A statistically significant association between trauma mechanism and presence of ligamentous knee injury was observed, with motor vehicle accidents exhibiting higher rates (34.78%) compared to falls from height (3.85%).

The high-energy trauma mechanism capable of causing pelvic and acetabular fractures may extend to the knee joint, leading to ligamentous injuries that may not be immediately evident due to the context of the more severe injuries. The initial priority in the treatment of these patients often focuses on hemodynamic stabilization and the management of life-threatening trauma [6,16]. In this context, a comprehensive knee evaluation may be secondary or limited by the patient’s inability to cooperate due to pain or altered consciousness.

To address this issue, we propose the integration of systematic knee evaluations into the trauma management protocol for patients with high-energy pelvic fractures. This could include a standardized secondary survey performed once the patient is hemodynamically stable, incorporating clinical tests such as the Lachman test or posterior drawer test to assess ligamentous integrity. We also propose performing a full knee examination under anesthesia when the patient is being treated for the pelvic or acetabular injuries. This offers an ideal time window to highlight any evident knee instability without patient pain levels to complicate the exam. While routine MRI screening for all polytrauma patients may not be feasible due to cost and resource constraints, a targeted approach—prioritizing patients with high-risk trauma mechanisms such as road traffic accidents—could optimize diagnostic yield.

In the longer term, the diagnostic oversight may have significant consequences. Patients who remain undiagnosed may experience prolonged disability, decreased quality of life, and reduced satisfaction with their overall treatment outcome. Moreover, the development of chronic knee instability or degenerative joint disease can necessitate more complex and invasive surgical interventions, increasing both healthcare burden and patient morbidity. Establishing early detection protocols could therefore serve as both a preventative and cost-effective measure.

The existing literature consistently regards pelvic ring and acetabular fractures as severe orthopedic traumas and, consequently, underscores the importance of peripheral limb evaluation in the emergency setting [17]. Pelvic and acetabular fractures can have a profound impact on patients’ medium- and long-term quality of life, especially in young patients, as many do not fully regain their pre-injury functional level in activities of daily living (ADL) and may experience mental health issues [21,22].

Multi-ligamentous knee injuries (MLKIs) represent a complex and severe condition affecting the knee joint, characterized by heterogeneous injury patterns caused mainly by high-energy trauma. Their complexity arises not only from their rarity but also from the wide spectrum of injury patterns and severity, each presenting unique challenges [23,24]. MLKIs involve partial or complete rupture of two or more of the major knee ligaments, including the ACL, PCL, medial collateral ligament (MCL), and lateral collateral ligament (LCL). These injuries range from isolated, closed ligamentous tears without additional structural damage to complex polytraumatic (PT) injuries. This variability requires a highly individualized approach to treatment, ranging from non-surgical methods, such as limb immobilization, to more commonly used surgical interventions, including acute ligament repair and reconstruction, the latter showing better functional and clinical outcomes [23,24,25]. Although PCL injuries are less common than ACL injuries, they are often involved in high-energy injury mechanisms and in the context of major pelvic and acetabular fractures [12,26,27]. Our findings are significant, suggesting that the injury mechanism is a crucial predictor of long-term knee complications, highlighting the importance of examining the knee joints prior to discharge, especially in those patients involved in road traffic accidents (RTAs). Demographic factors such as age and sex have a lesser influence on outcomes in this patient population.

Several studies, including the one by Levy et al., have shown that early surgical treatment significantly impacts medium- to long-term clinical and functional outcomes [28,29,30]. Therefore, failure to diagnose knee ligament injuries early could lead to chronic instability, loss of range of motion (ROM), persistent pain, and an increased risk of long-term osteoarthritis [25,31,32,33], which can have long lasting effects, especially in younger patients.

The results of our study highlight the challenge of promptly diagnosing concomitant ligamentous injuries in the context of high-energy pelvic and acetabular trauma. In the acute setting, the diagnostic focus prioritizes hemodynamic stability and management of life-threatening injuries. Our findings support the concept that systematic evaluation protocols, including secondary surveys and early imaging, may help avoid missed or delayed diagnoses of ligamentous injuries in this patient population.

The association between diagnostic delay and lower IKDC scores in patients requiring ligament reconstruction in this series suggests that timing of diagnosis may influence functional recovery. Prolonged undetected instability may contribute to progressive degenerative changes and future knee disability. Ensuring early recognition, whether through improved clinical vigilance or the use of adjunct imaging when suspicion exists, could optimize both surgical planning and rehabilitation outcomes.

Therefore, we recommend that all patients with high-energy pelvic ring and acetabular fractures undergo a thorough knee examination as soon as their clinical condition allows for it, ideally before hospital discharge. Furthermore, we also suggest that all patients who suffered pelvic ring or acetabular fractures as a consequence of an RTA should routinely be sent for an outpatient MRI at 3 months from hospital discharge, due to the higher likelihood of ligamentous knee injury.

Our study presents several limitations, including its retrospective nature, which may have restricted the completeness of knee assessment data at the time of initial trauma, and the relatively small sample size, which may limit the generalizability of the findings to a broader population. Additionally, the reliance on telephone interviews for data collection introduces the potential for recall bias, as patients may not accurately remember the onset or nature of their symptoms. The lack of routine MRI screening during the initial hospitalization further limits our ability to definitively quantify the prevalence of subclinical knee injuries at the time of trauma. Despite these limitations, our study provides valuable insights into the underdiagnosis of knee ligament injuries and highlights the need for prospective studies to validate and expand upon our findings. Prospective studies with standardized knee evaluation protocols are necessary to confirm our findings and better quantify the true prevalence of these injuries.

Moreover, the low incidence of these injuries has resulted in a shortage of large-scale, definitive clinical studies, leading to a lack of consensus on the key diagnostic and management strategies.

Consequently, future research directions may include prospective studies systematically evaluating knee injuries in patients with high-energy pelvic and acetabular fractures using both clinical examination and early MRI imaging. Additionally, further research could focus on the impact of early diagnosis and treatment of knee ligament injuries on long-term functional outcomes in this patient population. Future research should focus on developing standardized diagnostic algorithms that integrate clinical and imaging modalities to improve the detection of knee ligament injuries in polytrauma patients. Such studies could also explore the cost-effectiveness of routine MRI screening in high-risk groups and evaluate the impact of early intervention on long-term functional outcomes. Additionally, investigating the role of advanced imaging techniques, such as dynamic MRI or stress radiographs, could enhance diagnostic accuracy in patients with subtle or equivocal clinical findings.

## 7. Conclusions

In conclusion, our study suggests that undiagnosed knee ligament injuries may be more common than previously recognized in patients with surgically treated high-energy pelvic ring and acetabular fractures. Greater awareness of this potential association and the implementation of more comprehensive evaluation protocols could enhance the overall management of these patients and potentially improve their long-term outcomes.

Given the complexity and severity of multi-ligamentous knee injuries, early diagnosis and appropriate treatment are essential to minimize long-term disability, especially in the younger population. A systematic approach to evaluating ligamentous knee injuries in patients with pelvic and acetabular fractures is recommended to ensure prompt recognition and management of associated injuries, especially in those patients who were victims of RTAs.

Future studies should focus on developing diagnostic protocols to facilitate early identification of knee injuries in polytrauma patients, with the aim of improving functional outcomes and quality of life in the long term.

## Figures and Tables

**Table 1:** Telephone questionnaire questions.

Telephone questionnaire	
Trauma Mechanism	
Fracture Side	
Days of Hospital Stay	
In-Hospital Diagnosis of Knee Pathology?	
Knee Issues Post-Hospital Discharge?	If Yes, how was it diagnosed and when
Current Knee Issues	

**Table 2:** Patient Demographics.

Age (years)	55 (17–81)
Patient sex (male)	43 (72.9%)
Body Mass Index (kg/m^2^)	26.2 (3.3)
Patients with previous ligamentous reconstruction	1 (1.7%)

Age is presented as mean and range.

## Data Availability

All data can be made available upon reasonable request.
